# New Vaccines for Mammalian Allergy Using Molecular Approaches

**DOI:** 10.3389/fimmu.2014.00081

**Published:** 2014-03-14

**Authors:** Marianne van Hage, Gabrielle Pauli

**Affiliations:** ^1^Clinical Immunology and Allergy Unit, Department of Medicine Solna, Karolinska Institutet and University Hospital, Stockholm, Sweden; ^2^Faculty of Medicine, Strasbourg University, Strasbourg, France

**Keywords:** allergen-specific immunotherapy, Fel d 1, mammalian allergens, recombinant allergens, T cell peptides, vaccines

## Abstract

Allergen-specific immunotherapy (SIT) offers a disease specific causative treatment by modifying the allergen-specific immune response allowing tolerance to higher doses of allergen and preventing progression of allergic diseases. It may be considered in patients allergic to furry animals. Current mammalian allergy vaccines are still prepared from relatively poorly defined allergen extracts and may induce immediate and late phase side effects. Although the mechanisms of SIT are still not fully understood, the more recent approaches report different strategies to reduce both allergen-specific IgE as well as T cell reactivity. The availability of recombinant allergens and synthetic peptides from the mammalian species has contributed to formulating new allergy vaccines to improve SIT for furry animal allergy. The majority of studies have focused on the major cat allergen Fel d 1 due to its extensive characterization in terms of IgE and T cell epitopes and to its dominant role in cat allergy. Here we review the most recent approaches, e.g., synthetic peptides, recombinant allergen derivatives, different hypoallergenic molecules, and recombinant allergens coupled to virus-like particles or immunomodulatory substances as well as strategies targeting the allergen to Fcγ receptors and the MHC class II pathway using a new route for administration. Many of the new vaccines hold promise but only a few of them have been investigated in clinical trials which will be the gold standard for evaluation of safety and efficacy in allergic patients.

## Introduction

Allergen-specific immunotherapy (SIT) offers a disease specific causative treatment by inducing tolerance to the allergen and preventing progression of allergic diseases, for example development of asthma (1). Hence, patients who suffer from allergies where avoidance is not possible such as house-dust mite respiratory allergies are the prime candidates for SIT. Allergy to cats, dogs, or other furry animals is common in society and the prevalence of sensitization to pets has increased (2). Although the best treatment is avoidance, it is often difficult to realize as well as not being well accepted by the patients. Moreover allergens from furry animals, e.g., the major cat allergen Fel d 1 is equally present in houses without cats, in public places, public transport, day care centers, and school rooms. So the question of SIT may be considered. Allergen-specific immunotherapy for cat allergy has shown to be more favorable than for, e.g., dog allergy (3). This may depend on the standardization of cat extracts to Fel d 1, whereas dog extracts may vary greatly in terms of allergen content (4).

The mechanisms of SIT are still not fully understood. The clinical efficacy is associated with the production of allergen-specific blocking immunoglobulin (Ig)G antibodies and the generation of T regulatory cell (T regs) responses and secretion of immunoregulatory cytokines such as interleukin 10 (5, 6); however, current SIT involves drawbacks such as treatment persistence, due to the long treatment duration both with subcutaneous SIT and sublingual immunotherapy (7). Furthermore, SIT of today can induce adverse reactions ranging from mild symptoms to life-threatening anaphylaxis and even death (8).

Allergy vaccines of today are still made from crude allergen extracts, which are not well-defined. Since the structures of the most common allergen molecules are now available, well-defined recombinant and synthetic allergy vaccines can be generated allowing targeting specific mechanisms of allergic disease without increasing their allergenicity (9–11). Many of these new molecular approaches concern pollen allergens. It has been shown that SIT with the recombinant major birch allergen Bet v 1 is equally effective as SIT with birch pollen extract or natural purified Bet v 1 (12). In this review, we describe different approaches for new vaccines, e.g., recombinant allergen derivatives, different hypoallergenic molecules, and recombinant allergens coupled to virus-like particles or to immunomodulatory substances. Studies being available concern principally evaluation of the cat major allergen, either in animal models or in allergic patients.

## General Concepts to Increase Safety of Allergy Vaccines

Side effects are related to the production of allergen-specific IgE antibodies but can also be mediated by the allergen-specific T cells. Several studies during the last years have used an allergen-unrelated carrier molecule, which provides T cell help for the production of IgG antibodies but avoid the activation of allergen-specific T cells. This approach would reduce T cell mediated side effects. Different strategies to reduce both IgE as well as allergen-specific T cell epitopes have been reported. These approaches will lead to less IgE and T cell-mediated side effects (11, 13).

- Strategies targeting allergen-specific T cells by using T cell epitope-containing peptides (synthetic molecules produced by chemical synthesis), which give no IgE-mediated activation of effector cells.
- Strategies reducing allergen-specific IgE reactivity, but conserving T cell reactivity.
- Strategies based on carrier-bound fusion proteins using peptides. These allergen derived peptides are obtained from the IgE-binding sites of the allergen but contain no or reduced allergen-specific T cell epitopes and exhibit no or strongly reduced IgE reactivity. The peptides are fused to an allergen-unrelated viral carrier molecule and expressed as recombinant proteins. This technology was promoted by the Vienna group and a grass-pollen allergy vaccine based on carrier-bound peptides of the four major grass-pollen allergens is now under evaluation in a clinical phase II trial (11, 13–15).

Several other strategies for improving SIT have been described in the studies published within the last years: e.g., targeting the allergen to Fcγ receptors (16), targeting the MHC class II pathway (17), linking allergens to CpG-containing nucleotides (18, 19) or to carbohydrate-based particles (20), and co-administrating allergens with immunomodulatory substances such as vitamin D3 (21, 22). DNA vaccines favoring specific Th1 responses may also be used with different adjuvants such as CpG. mRNAs encoding allergens are another possible strategy, which would improve the safety of therapeutic nucleic acid-based vaccines. Moreover, different routes for SIT have been investigated using recombinant allergens especially sublingual, epicutaneous, and intra-lymphatic delivery of the allergen (23).

Table 1 provides a summary of these approaches that have been applied for furry animal allergen SIT. It is interesting to note that the majority of studies have focused on the major cat allergen Fel d 1 due to its dominant role in cat allergy and to its extensively characterization in terms of IgE and T cell epitopes.

**Table 1:** **Concepts and strategies for improving SIT for mammalian allergy**.

	Technologies	Specific approaches	Reference
Synthetic molecules	Peptides
		Mixture of 12 short peptides	Oldfield et al. (24)^a^
		Mixture of 7 short peptides	Worm et al. (25)^a^, Patel et al. (26)^a^
Recombinant molecules	Hypoallergens
	Mutants	Mus m 1	Ferrari et al. (27)
	Hybrids	Fel d 1 duplicated T cell epitopes and disruption of disulfide bounds	Saarne et al. (28)
	Random mutation	Fel d 1	Nilsson et al. (29)
	Peptide carrier fusion proteins
	Derived virus particles	Qß-Fel d 1	Schmitz et al. (30)
	Pre-S domain of hepatitis B	Fel d 1 peptides fused to Pre-S	Niespodziana et al. (31)
Conjugation with immunomodulatory molecules	Fcγ receptor	Truncated IgG Fcγ Fel d 1	Zhu et al. (16)
	Truncated invariant chain peptide + transactivator of transcription	MAT-Fel d 1	Martinez-Gomez et al. (17), Senti et al. (32)^a^
	Vitamin D3	Fel d 1-Vitamin D3	Jeffery et al. (33), Grundström et al. (22)
	Carbohydrate-based particles	CBP-Fel d 1	Neimert-Andersson et al. (20), Thunberg et al. (34)

*MAT, modular antigen transporter; CBP, carbohydrate-based particle*.

*^a^Clinical studies*.

## T Cell Peptides

T cell peptides were originally designed to modulate allergen-specific T cell responses without IgE-mediated activation of effector cells (35). Following preclinical studies (36), a series of clinical trials were performed to evaluate the safety and efficacy of two polypeptides from the major cat allergen Fel d 1. Initially, patients received a subcutaneous injection weekly for 4 weeks with an equimolecular mixture of the peptides (ALLERVAX CAT; Immunologic Pharmaceutical Corporation, Waltham, MA, USA, an equimolar combination of two 27-aminoacid synthetic peptides), 7.5, 75, and 750 μg, or placebo. Significant improvements in lung and nasal symptoms were observed in the high dose group but many adverse events occurred several hours after peptide injection (37). A subsequent multicenter study in which patients received eight injections of 750 μg of vaccine reported only modest clinical improvement and immediate and late side effects were observed (38). Further developments using a mixture of 12 short peptides comprising the majority of the T cell epitopes from Fel d 1 demonstrated that changes in the quality of life of the active treated group versus the placebo group, but isolated late asthmatic reactions were still observed (24). In a recent study, a new vaccine was developed consisting of a mixture of seven immunodominant peptides (ToleroMune cat^®^, also known as cat PAD, Circassia Ltd, Oxford, UK), which were selected on the basis of MHC-binding characteristics. This approach allowed reducing the number of peptides without substantially compromising population coverage. The safety and tolerability of the vaccine was evaluated in a phase IIa study (25) where cat-allergic individuals were given a single dose of the vaccine either intradermally or subcutaneously. Inhibition of the late cutaneous reaction was considered as a surrogate of clinical efficacy and 3 nmol was determined as the best concentration for intradermally desensitization. Treatment was performed in patients with cat-induced allergic rhinoconjunctivitis and in an environmental exposure chamber they were exposed to cat allergen before and after therapy. The clinical efficacy was observed after 18–22 weeks and 50–54 weeks after the start of the treatment and the highest dose of cat PAD (6 nmol 4 weeks apart, compared to 3 nmol 2 weeks apart) gave the greatest efficacy. The treatment effect was apparent on nasal and ocular symptoms and persisted 9 months later without any further retreatment (26). Other clinical studies should confirm these encouraging results.

T cell peptides containing major allergen epitopes have been generated from the primary structures of other mammalian allergens such as Can f 1 (39), Bos d 2 (40), and Equ c 1 (41) but the potential application of these molecules in allergic patients have not been investigated.

## Recombinant Hypoallergenic Derivatives

Hypoallergenic derivatives are characterized by a strongly reduced IgE reactivity. The IgE-binding epitopes of Fel d 1 have been modified through disruption of disulfide bonds and duplication of T cell epitopes. Three of the modified Fel d 1 derivatives displayed a strong reduction in allergenicity with 400–900 times lower IgE-binding capacity (hypoallergens) compared to wild-type Fel d 1 (28). The therapeutic capacity of the hypoallergen with the most reduced IgE reactivity was evaluated in a mouse model for cat allergy and by skin tests on cat-allergic patients. An induction of Fel d 1-specific IgG antibodies with capacity of blocking patients’ IgE to rFel d 1 and a reduction in airway responsiveness was noted. Furthermore, the hypoallergen showed a tendency of reduced SPT reactivity compared to rFel d in seven cat-allergic patients (42).

Other hypoallergenic mutants of rFel d 1 have been generated by the introduction of random mutated allergen sequences and the selection of derivatives with a maintained tertiary structure by phage display using IgE antibodies from cat-allergic patients. The mutants had a lower IgE-binding and T cell activation capacity and could induce strong IgG-anti Fel d 1 protective responses by mouse immunization experiments. Thus they should be good candidates for safe alternatives for SIT (29).

Furthermore, in search of a vaccine for mouse allergy a structure-guided single point mutation has been performed for Mus m 1, the major mouse allergen which is an urinary protein. This mutation induced a spatial rearrangement of aromatic side chains and a lower allergenic activity although the T cell reactivity was preserved (27).

## Peptide Carrier Fusion Proteins

One strategy to improve SIT has been to couple allergens to the bacteriophage Qß-derived virus-like particles. In mice, one injection of Qß coupled to Fel d 1 induced a Fel d 1-specific IgG response, and reduced anaphylactic reactions after rFel d 1 challenge (30). However, the chemical coupling process might be difficult to standardize for vaccine production.

Another carrier protein, the Pre-S domain of hepatitis B virus has been evaluated more recently (31). The cat hypoallergen vaccine was produced by fusion of Pre-S of hepatitis B to two non-allergenic Fel d 1 derived peptides. This approach has shown to eliminate both IgE-mediated and T cell mediated side effects. The T cell help comes from a Fel d 1-unrelated carrier protein, the Pre-S domain of hepatitis B virus, which contains antigenic sites for both B and T cells (43). The recombinant fusion protein exhibited more than 1000-fold reduction in allergenic activity compared with rFel d 1 (31). After immunization of mice and rabbits the fusion protein induced Fel d 1-specific IgG antibodies, which inhibited IgE-binding of cat-allergic patients to Fel d 1. In addition, the T cell responses in immunized mice were specific for Pre-S and very low for Fel d 1. This vaccine has to be further evaluated in clinical studies to confirm its promising qualities.

## Conjugation to Molecules with Immunomodulatory Functions

A chimeric human–cat fusion protein composed of a truncated human IgG Fc gamma 1 and Fel d 1 has been proposed for a new approach of SIT (16). This conjugate induced a dose dependent inhibition of Fel d 1 driven IgE histamine release from cat-allergic donors’ basophiles and from sensitized human cord-blood derived mast cells. The vaccine potential was also demonstrated in a mouse model for cat allergy (16, 44).

A new technology called modular antigen translocation (MAT) has been applied to Fel d 1 to enhance immunogenicity and safety of SIT. By attaching a truncated invariant chain peptide, and a transactivator of transcription peptide to allergens, they are converted into cytoplasmic proteins targeting the MHC class II pathway (17). MAT fusion of rFel d 1 has shown to enhance protective antibody and Th1 responses in mice, while reducing human basophil degranulation (17). A recent paper has described a phase I/IIa, randomized, placebo-controlled, and double-blind trial, using this construct by intra-lymphatic injections in cat dander allergic patients (20 patients were randomized) (32). The intra-lymphatic route has shown to reduce both the number of allergen injections as well as the allergen dose, improving both efficacy and safety of SIT (45). Three monthly injections with increasing doses of MAT-Fel d 1 elicited no adverse events and there was significant increase in allergen tolerance after nasal provocation. Regulatory T cell responses were stimulated and IL10 cytokine secretion and increased cat dander specific IgG4 production were observed. This clinical study represents the first immunotherapy trial with a recombinant cat allergen. It is also a major improvement over classical immunotherapy due to improved safety, low allergen doses, few injections, and a short treatment period. This promising vaccination approach has to be confirmed in larger patient studies and also by assessing efficacy on reduction of symptoms and medication use and long term follow-up.

1,25-Dihydrovitamin D3 has been shown to induce dendritic cells with tolerogenic properties, thus, increasing regulatory T cell responses (33). In an attempt to use this effect, VD3 was covalently coupled to rFel d 1 and tested in a mouse model for cat allergy (22). rFel d 1 VD3 was superior to rFel d 1 in reducing airway inflammation, and airway hyperresponsiveness, and in producing allergen-specific IgG blocking antibodies.

rFel d 1 has also been covalently coupled to carbohydrate-based particles (CBP) for targeting of dendritic cells and enhanced adjuvanticity in SIT (34). This approach was evaluated in a mouse model for cat allergy (20). CBP-rFel d 1 treated mice showed reduced features of allergic inflammation and increased allergen-specific IgM and IgG responses. In a prophylactic protocol it was also shown that CPB rFel d 1 prevents the induction of airway inflammation possibly through the induction of allergen-specific IgG and IgM and by a prolonged tissue exposure to rFel d 1 (34).

## Conclusion

Better characterization of recombinant allergens from mammalian species has contributed to formulate new allergy vaccines to improve SIT for patients allergic to furry animals. The identification of the major allergens in allergen sources is essential for generating vaccines, which may replace the natural allergen extract (11, 46). This explains in part why a majority of the new furry animal allergy vaccines have been restricted to the major cat allergen Fel d 1. Further studies are needed to point out which allergen component/s are needed for treatment of furry animal allergy, taking in account the great variability of commercial extracts regarding their allergen contents. This is especially true for dog allergens (4) where the panel of allergens so far is incomplete. Several studies presented here explore new concepts for improving the safety of SIT, by using different approaches and various technologies. However, only a few of them have been investigated in clinical trials, which will be the gold standard for evaluation of safety and efficacy in allergic patients.

## Conflict of Interest Statement

The authors declare that the research was conducted in the absence of any commercial or financial relationships that could be construed as a potential conflict of interest.
